# 

**Published:** 1881-02

**Authors:** 


### CONTENTS FOR FEBRUARY, 1881.

ORIGINAL COMMUNICATIONS.

ART. I.—Spasm of the Eye Muscles. By JULIAN J. CHISOLM, M. D. .......... 51
ART. II.—Some Observations on the Use of the Lancet. By RUFUS W. GRISWOLD, M. D. .......... 60
ART. III.—Case of Strangulated Scrotal Hernia. By PROF. HARVEY L. BYRD, M. D., Assisted by G. HALSTED BOYLAND, A. M., M. D. .......... 66
ART. IV.—Case of Facial and Palatine Paralysis and Loss of Equilibrium, Produced by a fall on the Head. By M. PUTNAM JACOBI, M. D. .......... 69

CORRESPONDENCE.

Medical Laws of Alabama .......... 74

EDITORIAL DEPARTMENT.

Provost of the University of Pennsylvania .......... 77
Planetary Perihelia of 1881 .......... 77
Apropos of the Figures 1881 .......... 78
A difference in the Count .......... 78
Snowden's Binaural Stethoscope .......... 78
Diphtheria, Muriate of Pilocarpin in .......... 79
Our Sincere Thanks .......... 80
To a New Subscriber .......... 80
Alabama State Laws regulating the Practice of Medicine .......... 81
Errata .......... 81
An American Physician in Germany .......... 82
Petroleum in Phthisis .......... 82
Bartholows Cartwright Lectures .......... 83
Trichinosis Cured .......... 83
Dr. L. P. Yandall .......... 83
Popular Science—A Proposed New Department .. 83

BIBLIOGRAPHICAL DEPARTMENT.

Practical Treatise on Diseases of Women .......... 84
Medical Heresies Historically Considered .......... 85
Rocky Mountain Health Resorts .......... 86
Chronic Bright's Disease in Children .......... 86
Third Annual Report of the Presbyterian Eye and Ear Charity Hospital of Baltimore .......... 86
A Spring Course of Anatomy and Operative Surgery .......... 87
The Physician's and Surgeon's Investigation .......... 88
The Medical annuls .......... 88
Transactions of the American Dental Association 88
Transactions of the Iowa State Dental Society .......... 88
Caries of the Superior Maxilla .......... 89
Anæsthesia .......... 89
Medical Progress .......... 89
The American Medical Bi-Weekly .......... 89

THERAPEUTIC PEARLS AT RANDOM STRUNG .......... 90–94

HYMENEAL, NECROLOGICAL .......... 95

EXCEPTA.

A Simple Explanation .......... 96
Bishops and Doctors .......... 96
Curiosities of Vital Statistics .......... 96
Borax in Hoarseness .......... 97
Physioligical Action of Conium Maculatum .......... 97
Chrysophanic Acid in Skin Disease .......... 98

